# Correction: Scleral appearance is not a correlate of domestication in mammals

**DOI:** 10.1186/s40851-024-00242-z

**Published:** 2024-10-28

**Authors:** Kai R. Caspar, Lisa Hüttner, Sabine Begall

**Affiliations:** 1 Institute of Cell Biology, Heinrich Heine University Düsseldorf, Düsseldorf, Germany; 2 Department of General Zoology, University of Duisburg-Essen, Essen, Germany; 3 Department of Game Management and Wildlife Biology, Faculty of Forestry and Wood Sciences, Czech University of Life Sciences, Praha, Czech Republic

**Zoological Letters (2023) 9:12**

10.1186/s40851-023-00210-z

Following publication of the original article, it came to the attention of the authors that there were errors in Supplementary File 1. Namely, some of the data concerning *mean scleral brightness* and *HC* entries for the genus *Mustela* were incorrect. The species-level value for *mean scleral brightness* in domesticated *Mustela* was found to be 128.3 (rather than 107.9) and was 146.93 (rather than 124.6) in the non-domesticated form (*M. putorius* / *eversmanni*). Regarding the species-level HC value, this needed to be adjusted to 133.34 (in place of 132.67) for non-domesticated *Mustela* but had been correctly reported for the domesticated form of *Mustela*. These errors affected the analyses presented in the article, which were all based on the erroneous dataset in question, as well as the figures, which, as a result of the errors, were inaccurate with respect to data points corresponding to *Mustela*.

The article original article has been updated to correct the errors in question. In this regard, the authors note that the errors were relatively minimal and that the correcting thereof does not affect the interpretation of the paper’s findings. The results of phylogenetic paired *t*-tests remain almost unchanged (comparison of domesticated vs. non-domesticated forms -  *mean scleral brightness* data: *t* = 0.96, *p* = 0.36; *HC*: *t* = 0.95, *p* = 0.36) and the effects of eye size on scleral brightness in the initial PGLS model that included the entire species sample remain non-significant (*p* > 0.325). The authors thank you for reading this erratum and apologize for any inconvenience caused.

## Electronic supplementary material

Below is the link to the electronic supplementary material.

Supplementary Information 1

